# Confirmed Ceylon krait (*Bungarus ceylonicus*) envenoming in Sri Lanka resulting in neuromuscular paralysis: a case report

**DOI:** 10.1186/s13256-017-1503-0

**Published:** 2017-11-24

**Authors:** Chamara Dalugama, Indika Bandara Gawarammana

**Affiliations:** Department of Medicine, University of Peradeniya, Peradeniya, Sri Lanka

**Keywords:** Snakebite, Ceylon krait, *Bungarus ceylonicus*, Sri Lanka, Neuromuscular paralysis

## Abstract

**Background:**

Ceylon krait (*Bungarus ceylonicus*) is a venomous elapid snake endemic to Sri Lanka. It inhabits shaded home gardens and forests in the wet zone of Sri Lanka and might creep into houses in the night. Despite frequent encounters with humans, reports of envenoming are very rare.

**Case presentation:**

We report a case of a 26-year-old Sri Lankan Sinhalese man with confirmed Ceylon krait envenoming presenting with bilateral partial ptosis, ophthalmoplegia, facial muscle weakness, and dysphagia. Single fiber electromyography and repetitive nerve stimulation confirmed neuromuscular paralysis. He was administered polyvalent anti-venom serum immediately following admission without a prompt clinical response. Complete recovery was observed 3 days following the bite.

**Conclusions:**

Because of the rarity of envenoming, precise and detailed information on the clinical manifestations following envenoming is lacking. However, Ceylon krait bite can be potentially fatal; so, treating physicians should be aware of species identification, habitat, and biting habits and clinical presentation of envenoming of Ceylon krait. This case report adds knowledge to the existing limited literature available on Ceylon krait envenoming; a rare but potentially fatal clinical entity.

## Background

Ceylon krait (*Bungarus ceylonicus*) is a venomous elapid snake endemic to Sri Lanka. Its general morphological features are similar to the common krait, but in contrast there are 15 to 25 distinct single white crossbars on the dorsal aspect which run across its belly. The snake is mainly distributed in the hilly wet zone. It frequents habitats near human dwellings and might creep into houses in the night. It is not vicious and tends to escape in captivity [1]. Despite frequent encounters with humans, reports of envenoming are very rare and limited to six in the literature, which include one case of fatal envenoming and another case of neuromuscular paralysis with full recovery [2–6].

## Case presentation

A 26-year-old Sri Lankan Sinhalese man presented to the Toxicology Unit of our teaching hospital following a snakebite. He was an apprentice under a herpetologist. He presented following a bite by Ceylon krait (*Bungarus ceylonicus*). The species was accurately identified by the patient himself and by the herpetologist and confirmed by the authors. He sustained the bite to the dorsal aspect of his left hand around 7 p.m. while handling the snake. He presented to the hospital at 9 p.m. On admission he was conscious and rational. He complained of double vision and painful eye movements. He complained of difficulty in swallowing mainly for liquids with nasal regurgitation. He had colicky abdominal pain. He had passed urine after admission and denied red/Coca-Cola colored urine. He did not have any bleeding manifestations.

On examination his higher functions were intact. His pulse rate was 78 beats per minute with blood pressure of 120/70 mmHg and the rest of the cardiovascular system examination was unremarkable. His respiratory rate was 16 cycles per minute and the rest of the respiratory system examination was unremarkable. His abdomen was soft and non-tender. He had barely visible fang marks on the dorsum of his hand with no local reaction. He had bilateral partial ptosis with complex ophthalmoplegia. Palatal movements were sluggish. His neck muscle power was weak and cough reflex was diminished. He had tongue fasciculations. Although he complained of generalized myalgia, his upper limb and lower limb muscle tone, power, and reflexes were normal.

His whole blood clotting time was less than 20 minutes. His complete blood count hemoglobin was 11.6 g/dL, white cell count was 8.9 × 10^6^, and platelet count was 210 × 10^3^. His renal functions and transaminases were within the normal limits. His prothrombin time (PT) was 12 seconds (control, 13 seconds) and activated partial thromboplastin time (APTT) was 30 seconds (control, 31 seconds).

**Fig. 1:**
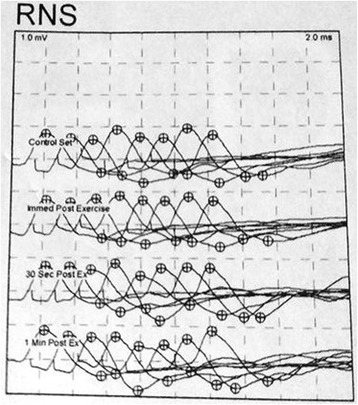
Repetitive nerve stimulation (RNS) showing the decrement-increment response

Single fiber electromyography (SFEMG) was performed on the third day after the bite. SFEMG was done to test his facial nerve and orbicularis oculi muscle. More than 20 fibers were sampled. Median jitter was abnormally delayed with a value of 43.2 ± 16.6 and mean jitter was 34.7 ± 17.9. Reference upper normal limit of the median jitter was 12.4. Blocking was present and 56% of fibers were abnormal (normal limit 20%). Repetitive nerve stimulation (RNS) showed the decrement-increment response (Fig. 1). So the electrodiagnostic findings were consistent with a neuromuscular junction function abnormality.

On admission he was administered polyvalent Indian anti-venom (which is the only available anti-venom at the moment in Sri Lanka) to which he hardly made any response and his neurological deficit peaked on the following day. He was closely monitored in the Toxicology Intensive Care Unit with regular clinical observations. Daily serum electrolyte, clotting profile, serum creatinine, and complete blood count were done and they were within the normal range. He was on liberal orally administered fluids with a daily maintenance of 3 liters and he maintained satisfactory urine output. Gradual improvement was noticed from the third day onwards. He did not undergo Tensilon (edrophonium) testing or a neostigmine trial as he was improving over the days. On the fifth day his neurological deficit was fully reversed and he was discharged. Repeat SFEMG and RNS performed 2 weeks later were essentially normal records.

## Discussion

Confirmed *Bungarus ceylonicus* encounters are extremely rare in the literature and confined to a very few cases. The first reported case dates back to 1908, a case report describing the death of a laborer from a confirmed Ceylon krait bite [2]. Then De Silva *et al*. reported three confirmed cases of Ceylon krait bites [3–5].

One such case of Ceylon krait envenoming reported in 1993 in Sri Lanka [5] described the case of a 30-year-old woman who presented to health care with a patient delay of 6 hours and had bilateral ptosis with respiratory muscle weakness. As a complication of traditional treatment she had aspirated as well. Unfortunately she developed an anaphylactic reaction to anti-venom. Although successfully resuscitated she remained deeply unconscious in a ventilator and died after 90 hours.

Another two cases were reported recently in literature [6]. A 25-year-old manual laborer presented with a Ceylon krait bite that had been provoked by contact of the foot. The live snake was brought and identified as Ceylon krait. He did not have any symptoms and all blood investigations were within normal range; he was observed for 3 days and in the absence of any symptoms, was reassured and discharged. In the second case a 58-year-old estate worker was bitten by Ceylon krait while sleeping on the floor of her compound. The snake was killed and brought along with the patient and it was identified by the authors. She had bilateral ptosis, external ophthalmoplegia, and facial muscle weakness. She could not open her mouth completely, nor could she protrude her tongue. She was administered anti-venom serum immediately. She made a full recovery from neuromuscular paralysis and was discharged on day four.

Neuroparalysis is the most common manifestation of common krait envenoming which includes ptosis, external ophthalmoplegia, respiratory paralysis, paralysis of neck flexors, dysphagia, and weakness of limbs [7, 8]. These manifestations can occur very rapidly and may be very severe needing prolonged periods of mechanical ventilation. We report a case of Ceylon krait bite with neuromuscular paralysis similar to two cases of envenoming by Ceylon krait reported in the literature. All had bilateral ptosis, ophthalmoplegia, and facial weakness. In our case, the above neurological observations were confirmed by SFEMG and RNS. But only one case resulted in respiratory failure following Ceylon krait bite needing mechanical ventilation.

Abdominal pain is a prominent clinical feature following common krait envenoming [9]. Our patient had colicky abdominal pain on presentation following Ceylon krait bite. Abdominal pain was not previously documented following a Ceylon krait bite.

The anti-venom available in Sri Lanka is imported from India where it is considered effective against cobra (*Naja naja*), Indian krait (*Bungarus caeruleus*), Russell’s viper (*Daboia russelii*), and saw-scaled viper (*Echis carinatus*) venoms. However, it has no proven efficacy for venom of the Ceylon krait. It was used in 1993 for a confirmed case of Ceylon krait envenoming and the patient developed a severe anaphylactic reaction and died [5]. Another case of Ceylon krait bite with neuromuscular paralysis was administered anti-venom immediately following admission to which the patient developed a mild reaction and there was no prompt response following anti-venom, although the patient made a gradual but full recovery [6]. In our case the patient was given anti-venom immediately after admission. No reaction to anti-venom serum was observed. However, neuromuscular paralysis was worse on the following day compared to admission but he made a full recovery over 3 days without additional therapy with anti-venom serum. Therefore, the imported venom is unlikely to be effective for native Sri Lankan species; however, based on very few case reports of Ceylon krait bites such a conclusion is difficult to reach. According to the available data, three patients, including the index case in this case report, received anti-venom but neurotoxicity progressed despite the anti-venom and late spontaneous recovery was seen in two cases and one died following an allergic reaction.

Neuromuscular paralysis in Ceylon krait bite is probably due to presynaptic neurotoxins (β-bungarotoxins) in the venom which start damaging the motor nerve terminal irreversibly, which could happen before anti-venom is given [10]. Once the neurotoxins start to damage the motor nerve terminal, the process is irreversible and heals with the natural recovery of the motor nerve terminal in 3 to 5 days; this means that even if the anti-venom is fully efficacious, still it would not be clinically effective. The presynaptic action is irreversible and is the reason that once neuromuscular paralysis develops it is not reversed with anti-venom, which is observed among other krait bites as well [11]. So although data are limited this could be an alternative explanation of the finding that anti-venom does not reverse the neurotoxicity immediately in Ceylon krait bites.

Indian polyvalent anti-venom may not be effective for Ceylon krait envenoming in Sri Lanka, because it may not neutralize the Ceylon krait venom and presynaptic neurotoxicity may be irreversible. Taking into account the very limited data available in the literature, one might have to consider the theoretical possibility of anti-venom being able to clear circulating free venom and preventing progression of neuromuscular dysfunction. So, management of a Ceylon krait bite still remains an enigma. Therefore, the safety and benefits of anti-venom need to be weighed along with the clinical status of the patient before deciding on anti-venom therapy.

## Conclusions

Ceylon krait is an endemic elapid in Sri Lanka that frequents habitats near human dwellings of the hilly wet zone and frequently intrudes into houses in the night. However, bites and cases of envenoming are extremely rare and limited to six cases in the last century. Because of the rarity of envenoming, precise and detailed information on clinical manifestations following envenoming is lacking. Very limited information is available on venom profile and molecular biology. The Ceylon krait bite can be potentially fatal; so, treating physicians should be aware of species identification, habitat, and biting habits and clinical presentation of envenoming of Ceylon krait. This case report adds knowledge to the limited literature available on Ceylon krait envenoming; a rare but potentially fatal clinical entity. Ideal management of a Ceylon krait bite is still debated and remains an enigma; it needs to be decided on an individual basis depending on the clinical status of the patient and the safety and benefits of the available treatment.
